# Correction: Jiang et al. Identification of Two Novel Linear B Cell Epitopes on the CD2v Protein of African Swine Fever Virus Using Monoclonal Antibodies. *Viruses* 2023, *15*, 131

**DOI:** 10.3390/v18010077

**Published:** 2026-01-06

**Authors:** Wenting Jiang, Dawei Jiang, Lu Li, Jiabin Wang, Panpan Wang, Xuejian Shi, Qi Zhao, Boyuan Liu, Pengchao Ji, Gaiping Zhang

**Affiliations:** 1 College of Veterinary Medicine, Henan Agricultural University, Zhengzhou 450046, China; jiang18860354181@163.com (W.J.); jiangdawei1010@126.com (D.J.); li18338215039@163.com (L.L.); wangjiabin0923@163.com (J.W.); wangpan11191001@163.com (P.W.); m18703610387@163.com (X.S.); m18838980392@163.com (Q.Z.); aka101lby@163.com (B.L.); 2 International Joint Research Center of National Animal Immunology, Zhengzhou 450046, China; 3 Longhu Laboratory, Zhengzhou 450046, China; 4 Henan Engineering Laboratory of Animal Biological Products, Zhengzhou 450046, China

## Error in Figure

In the original publication [1], there was a mistake in Figure 2 as published. Specifically, in Figure 2B (caption: IFA test results of mAbs 6C11 and 8F12 against CD2v), the subfigure labeled Panel mAb 8F12 (intended to depict IFA test result of mAb 8F12 against CD2v) was mistakenly replaced with the content of Panel mAb 6C11 when compiling the multi-panel composite figure. Importantly, the original caption for Figure 2 (including the descriptions for both Panel mAb 8F12 and Panel mAb 6C11) is fully accurate and requires no modification. The corrected Figure 2 appears below. The authors state that the scientific conclusions are unaffected. This correction was approved by the Academic Editor. The original publication has also been updated.

## Figures and Tables

**Figure 2:**
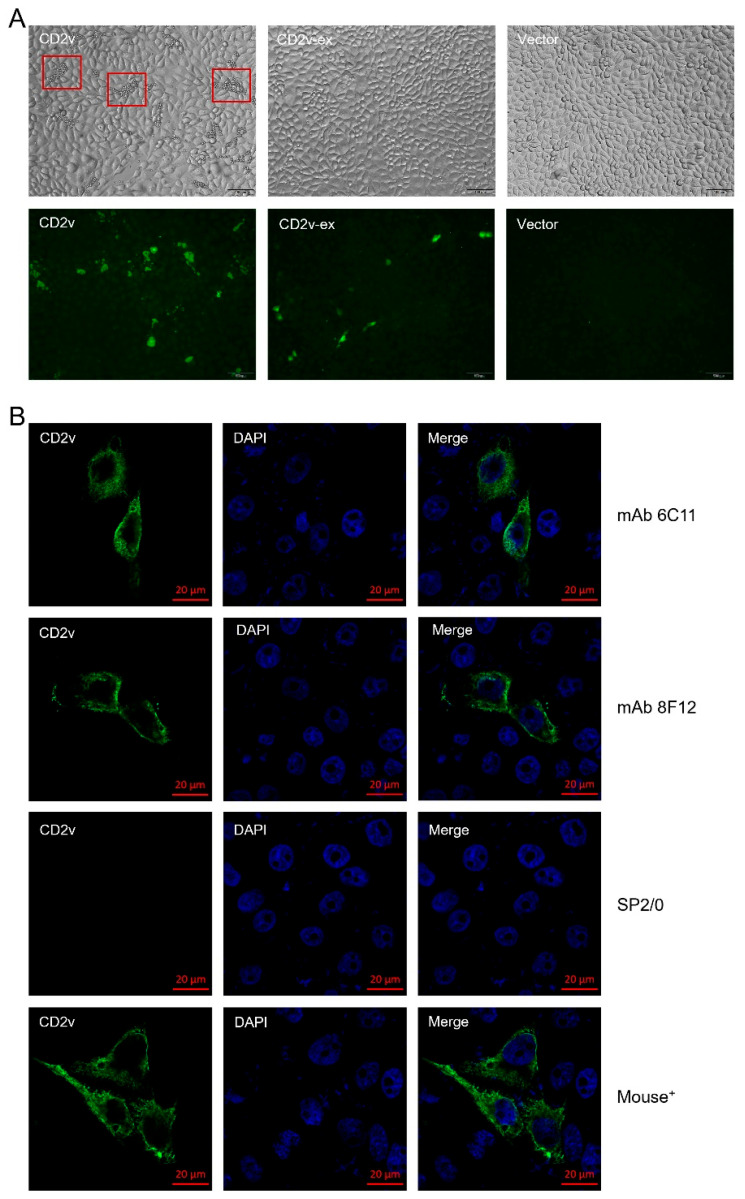
Localization of CD2v protein in transfected cells. (**A**) HAD test results of CD2v protein in transfected PK-15 cells. (**B**) IFA test results of mAbs 6C11 and 8F12 against CD2v. SP2/0 cell supernatants and anti-CD2v-ex mouse polyclonal antibodies (Mouse^+^) were used as negative and positive controls, respectively.
